# Exploring the role of cell cycle regulation in human mature adipocyte dedifferentiation

**DOI:** 10.3389/fcell.2025.1547836

**Published:** 2025-05-08

**Authors:** Giada Ostinelli, Marie-Frédérique Gauthier, Nathalie Vernoux, Emilie Bernier, Tristan Dubé, Simon Marceau, Stéfane Lebel, Marie-Ève Tremblay, André Tchernof

**Affiliations:** ^1^ Institut Universitaire de Cardiologie et Pneumologie de Québec-Université Laval, Québec, QC, Canada; ^2^ École de Nutrition, Université Laval, Québec, QC, Canada; ^3^ Axe Neurosciences, Centre de Recherche du CHU de Québec-Université Laval, Québec, QC, Canada; ^4^ Division of Medical Science, University of Victoria, Victoria, BC, Canada

**Keywords:** dedifferentiation, dedifferentiated fat cells, DFAT, liposecretion, cell cycle

## Abstract

**Background:**

Dedifferentiated fat (DFAT) cells have been used in regenerative medicine due to their multipotent potential. According to the literature, the process of adipocyte dedifferentiation is characterized by liposecretion which results in a fibroblastlike, proliferating cell population, with increased expression of genes related to cell cycle. A number of pathways have been implicated in the process, but the role of the cell cycle in adipocyte dedifferentiation has yet to be investigated. Here we characterize the process of liposecretion, the cellular features of DFAT cells and the role of the cell cycle.

**Methods:**

Primary adipocytes and adipocyte-derived pluripotent cells (APC) were isolated from human adipose tissue and mature adipocytes were dedifferentiated in ceiling culture. The intracellular organization of DFAT and APC were compared using transmission electron microscopy (TEM), and the changes of intracellular lipid content over time were tracked with Oil Red O. Finally, we tested whether liposecretion is a cell cycle-dependent phenomenon by cultivating mature adipocytes in ceiling culture with or without four different inhibitors of the cell cycle (AraC, Irinotecan, Vincristine and RO-3306).

**Results:**

DFAT cells were enriched in intracellular lipids, which are stored in small lipid droplets. In addition, liposecretion, which characterizes mature adipocyte dedifferentiation, is characterized by the rapid secretion of a large lipid droplet that is coated by a membrane. This phenomenon seems to be hindered by the presence of cyclin dependent kinase 1 (CDK1) inhibitor RO-3306.

**Conclusion:**

Both human adipose tissue depots undergo dedifferentiation *in vitro*, but visceral adipose tissue DFAT cells retain more lipids than subcutaneous-derived DFAT cells. Liposecretion is characterized by the rapid ejection of a membrane-wrapped lipid droplet. This phenomenon is dependent on CDK1 and likely relies on the presence of integrin-mediated cellular adherence.

## Background

The phenomenon of adipocyte dedifferentiation has been first reported by Sugihara and collaborators in 1986, where in ceiling culture, mature adipocytes become fibroblast-like cells expressing a stem-cell signature (Sugihara et al., 1986). Authors later suggested that mature adipocytes are not cells found in a quiescent state, but rather can enter the cell cycle and divide (Sugihara et al., 1987). In this context they described two types of cell division, differing by the fate of the intracellular lipid droplet found inside unilocular adipocytes. Indeed, they suggested that either the unilocular lipid droplet leaves the mature adipocyte by asymmetrical cell division, i.e., generating an adipocyte and a fibroblast as daughter cells; or the unilocular adipocyte becomes multilocular and the intracellular lipid droplets are split evenly between the two daughter cells (Sugihara et al., 1987).

Research accomplished in the last decade allowed a better understanding of adipocyte dedifferentiation. Maurizi and collaborators first identified the phenomenon characterizing mature adipocyte dedifferentiation in ceiling culture as the secretion of a large intracellular lipid droplet, coated by a three-layered membrane, a process that was named “liposecretion” (Maurizi et al., 2017). We additionally contributed to the study of liposecretion by using live-cell time-lapse microscopy and confirmed that mature adipocyte rapidly secrete their intracellular lipid droplet, becoming fibroblast-like cells, named dedifferentiated fat (DFAT) cells (Côté et al., 2019a). The originating DFAT cell shows expression of genes associated with the cell cycle and cell regeneration (Côté et al., 2017a) and a number of groups have already used them as a source for transplant and regenerative medicine [reviewed in (Côté et al., 2019b)]. Current literature indicates that DFAT cells have similar proliferative abilities compared to adipose tissue-derived progenitor cells (APC), as well as adipogenenic, osteogenic and chondrogenic potentials (Tansriratanawong et al., 2020).

The mechanisms that trigger adipocyte dedifferentiation are a matter of discussion in the field. The autophagy axis has been recently suggested to participate in adipocyte dedifferentiation, as the incubation of 3T3-L1 adipocytes with bafilomycin A1 (an autophagy blocker) prevented dedifferentiation (Pan et al., 2021). Because DFAT cells express a significant number of extra-cellular matrix (ECM) genes (Côté et al., 2017a), and in particular collagen, Côté and collaborators suggested a role for transforming growth factor β (TGFβ), a known inhibitor of adipogenesis and a strong contributor to ECM remodeling (Côté et al., 2017b). Similarly, recent literature proposes ECM as the triggering signal (Karanfil et al., 2023; Liu et al., 2022). On the other hand, because this phenomenon is seen in the involuting mammary gland of lactating mice, other authors suggested that hormonal signals might be at the origin of the phenomenon (Wang et al., 2018). Because the proliferative ability of DFAT cells has been consistently reported in the literature (Côté et al., 2019b; Huang et al., 2022; Tanimoto et al., 2022; Tansriratanawong et al., 2020), and because a number of genes and markers related to mitosis and the cell cycle are increased in DFAT cells (Côté et al., 2019a; Côté et al., 2017a), we tested whether cell cycle progression was required for liposecretion. The main objective of this study was to characterize the process of liposecretion and the cellular features associated with the originating DFAT cell. We designed our study using primary human cells isolated from adipose tissue samples from bariatric surgery donors.

## Methods

### Mature adipocyte and APC isolation

Men and women undergoing bariatric surgery were recruited through the Quebec Heart and Lung Institute’s tissue Biobank and provided written, informed consent. This project was approved by the Institute’s Ethics Committee (2018-2951). Fresh adipose tissue samples were collected from a total of 12 women (age: 49.8 ± 12.2 years, body mass index: 43.0 ± 6.6 kg/m^2^) and 5 men (age: 49.2 ± 12.6 years, body mass index: 46.1 ± 7.0 kg/m^2^). Samples (1 g–5 g) from either the abdominal subcutaneous adipose tissue (SAT) compartment or the greater omentum (VAT) were taken at the time of surgery and collagenase digestion was initiated within the hour. Briefly, before digestion, tissue was weighted and for each gram of adipose tissue, 4 mL of collagenase type I diluted in Krebs-Ringer-Henseleit (KRH) buffer (final concentration 350 units/mL) was used. Tissues were placed in a dry shaking incubator for 45 min at 37°C and 60°strokes/minute as per a modified version of the Rodbell protocol (Rodbell, 1964). The digested adipose tissue was then filtered through a nylon mesh and the floating phase (i.e., containing mature adipocytes) was washed three times with a total volume of approximately 15 mL KRH buffer.

### Mature adipocyte culture

Mature adipocytes were cultivated in ceiling culture in a 6-well plate with a plastic bushing on which a microscope glass slide was delicately placed (Côté et al., 2019a; Lessard et al., 2015a). Matures adipocyte medium (20% MAM; DMEM-F12 supplemented with 20% calf serum, 2.5 μg/mL Amphotericin B and 50 μg/mL gentamicin) was added to a volume so that the cells would be immersed but the glass slide on which they adhere would not float on the surface.

### APC culture

Following isolation, APC were centrifugated at 1,200 rpm for 5 min and resuspended in preadipocyte growth medium (10% PGM; DMEM-F12 supplemented with 10% calf serum, 1% penicillin-streptomycin, 17 µM pantothenic acid, 33 µM biotin, 100 µM ascorbic acid and 2.5 μg/mL amphotericin B). The suspension was filtered through a 75 μm nylon mesh, then cells were centrifugated again, resuspended in medium and plated. Medium was changed every 2–3 days and cells were passaged once they reached 80% confluence.

### Transmission electron microscopy (TEM)

Briefly, mature adipocytes in ceiling culture and APC were cultivated in an ACLAR-coated coverslip. Cells were then fixed in 2% paraformaldehyde and 2% glutaraldehyde for 2 h at room temperature and then embedded in 4% agarose. Agarose-embedded cells were cut and processed post-fixation as described in (Savage et al., 2020). Briefly, sections were incubated in 2% osmium tetroxide and 1.5% potassium ferrocyanide for 1 h, followed by incubation in 1% thiocarbohydrazide for 20 min, and further incubated in 2% osmium tetroxide. Subsequently, samples were dehydrated and incubated with Durcupan resin (Electron Microscopy Sciences, Hatfield, PA, United States) overnight at room temperature. Thin sections were cut with a diamond knife on the vibratome at ∼73 μm thickness, and were then collected on mesh grids and imaged with an FEI Tecnai Spirit G2 transmission electron microscope operating at 80 kV and equipped with a digital camera ORCA-HR (10 MP; Hamamatsu).

### Oil Red O staining

Mature adipocytes cultured in ceiling culture or APC grown on a glass slide, were washed with PBS and then fixed with 1 mL formalin 10% for 15 min. Slides were then washed three times before incubation with Oil Red O staining (2 mg/mL) for 2 h at room temperature. Excess staining solution was washed with water before mounting the microscope slides. A 0.12 μm secure seal spacer, in addition to a glycine mounting medium, were used to mount the microscope slides. Photos (10 per slide) were acquired using an Olympus motorized inverted research microscope IX81 (Olympus Corporation, Tokyo, Japan) using an Evolution™ QEi Camera (Media Cybernetics, Rockville, Maryland) and analyzed in ImageJ.

### Time lapse experiments

For membrane labelling, mature adipocytes were transduced using CellLight® Lck-kinase GFP (Molecular Probes, Life Technologies, Eugene, Oregon, United States) using BacMam 2.0 technology. After isolation of the mature adipocyte-containing fraction, adipocytes were treated with BacMam 2.0 reagents reaching a final concentration of 1:10. Cells were then incubated at 37°C and 5% CO_2_ for 15 min. Following treatment, cells were plated and time-lapse microscopy was started the following day. To test the effects of cell cycle inhibitors (see following section), isolated mature adipocytes were cultured in 20% MAM for 3 days to allow mature adipocyte adherence. After that, media was changed and cell cycle inhibitors were added at the following concentrations: 0.5 μM AraC, 2 nM Vincristine, 0.5 μM Irinotecan and 5 μM RO-3306. 0.05% DMSO was used as the vehicle for Irinothecan and RO-3306. 20% MAM was used as control for AraC and Vincristine. Ceiling culture was followed under the microscope. For both time-lapse experiments culture conditions (temperature, humidity, CO_2_) were kept constant and live images were taken on the Zeiss Axio Observer Z1 Miscrocope using Axiocam 506 (Zeiss, Oberkochen, Germany). Photos were taken every 30 min for approximately 120 h in at least two Z positions with a 10x LD-A-Plan objective to TL phase and GFP. Images were analyzed using Zen Blue lite 2.3 Digital Imaging Software. The number of liposecretion events was manually counted and normalized over the total number of adipocytes present in the frame. To alleviate bias in the identification of liposecretion events, counting was performed by the same investigator (MFG) throughout all experiments. Events were computed as fold over control (AraC and Vincristine diluted in water) or fold over vehicle (RO-3306 and Irinotecan diluted in DMSO).

### Cell cycle inhibition

To block the cell cycle in four different checkpoints, we used four cell cycle inhibitors at multiple concentrations. AraC (i.e., Cytarabine) a cytosine competitor blocking the cell in the S phase of mitosis was used at concentrations of 10 μM, 5 μM, 1 μM, 0.5 μM, and 0.05 μM (Côté et al., 2019a; Momparler, 2013). Vincristine, which impedes microtubule assembly, blocking the cell in the M phase of mitosis was used at concentrations of 2 μM, 1 μM, 20 nM, 2 nM, and 0.2 nM (Côté et al., 2019a). Irinotecan a topoisomerase I inhibitor, which blocks the cell at the end of phase S checkpoint was used at concentrations of 0.5 μM, 0.1 μM, and 0.01 μM (Philippart et al., 2000; Lagadec et al., 2008), and finally, RO-3306 the cyclin D kinase 1 (CDK1) inhibitor, blocking the cell between G2 and the M phase was used at concentrations of 5 μM, 1 μM, and 0.5 μM (Vassilev, 2006; Voets et al., 2015; Swadi et al., 2019; Jang et al., 2014).

To establish the concentration and the time of exposure that would be the least toxic for the cells, we investigated the effects of each inhibitor on APC. Each experiment was repeated at least three times with APC isolated from a different tissue donor. The concentration for time-lapse microscopy on mature adipocytes was determined using APC and based on cellular proliferation, toxcitity, viability (WST-1), and colony forming unit (CFU) assay. The experiments and their associated results are listed in Supplementary data. Results gathered from these experiments showed that cellular toxicity becomes evident after 96 h incubation, regardless of the drug used. In addition, deviations from the concentrations chosen could be inefficient in stopping cellular proliferation or cause increased apoptosis (see Supplementary Material).

### Statistical analysis

Statistical analyses were performed using R version 4.3.1 (R Core Team, 2021). Assumptions were tested using packages *DescTools* (version 0.99.57) and *stats* (version 4.3.1). The incorporation of Oil Red O staining between APC, DFAT SAT and DFAT VAT was compared using planned contrast ANOVA, while the differences across days were tested *post hoc* following Bonferroni correction. Finally, the effect of cell cycle inhibitors on liposecretion was tested using Kruskall-Wallis non-parametric test followed by Dunnett’s test for comparing several treatments with a control. Graphs were generated using *ggplot* (version 3.5.1).

## Results

**FIGURE 1:**
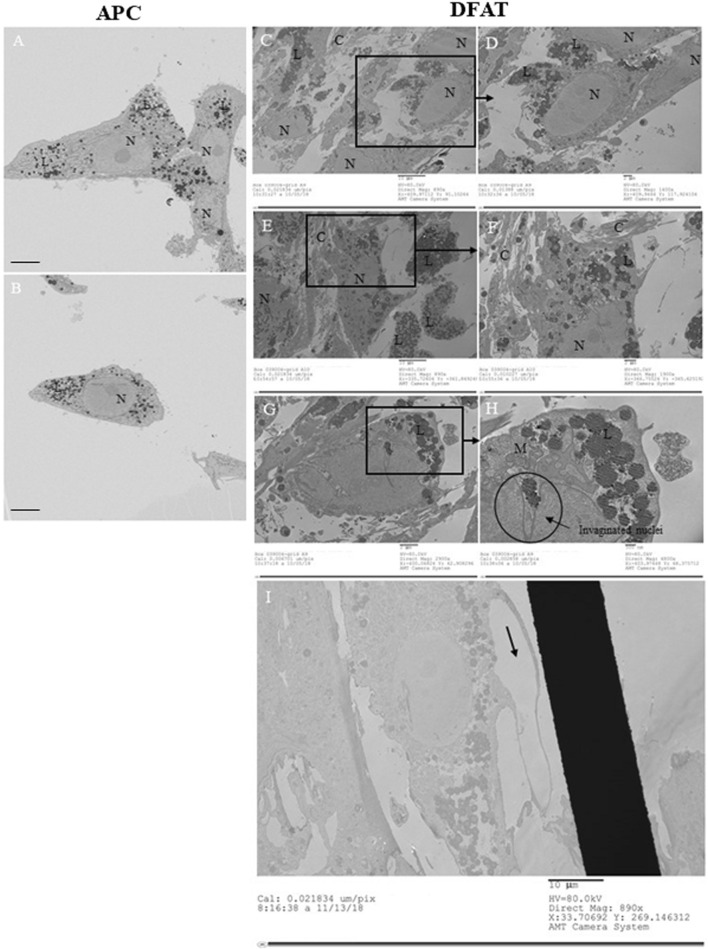
Transmission Electron Microscopy. Adipocyte-derived pluripotent cells (APC) in culture **(A, B)** Dedifferentiated fat (DFAT) cells cultivated on ACLAR after 7 days of ceiling culture **(C–I)**. Abbreviations: N = nuclei; C = collagen; M = mitochondria; L = lipid droplets.

**FIGURE 2:**
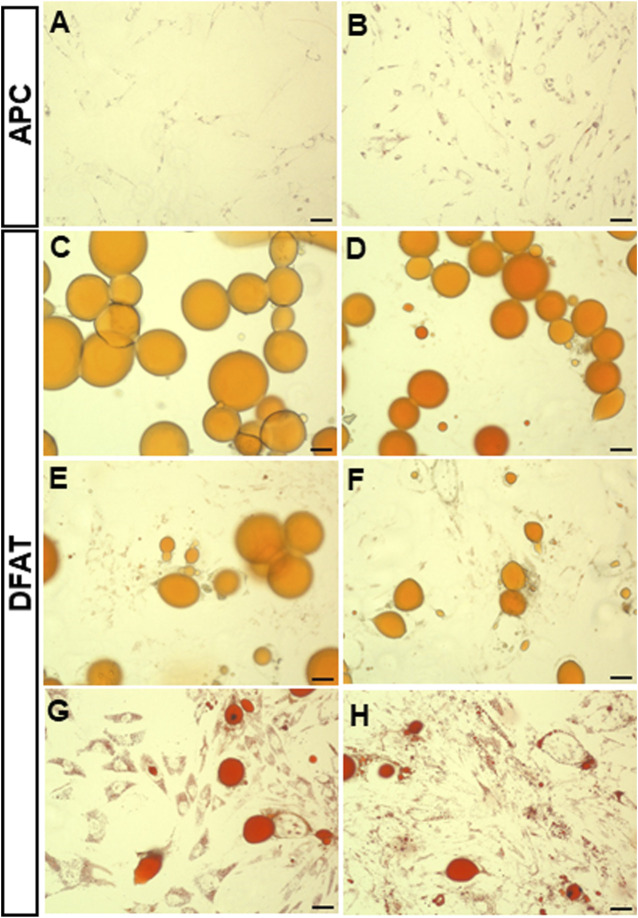
Oil Red O staining of dedifferentiating mature adipocytes and APC. Representative field of SAT **(A)** and VAT **(B)** APC and mature adipocytes **(C–H)**. Fields show day 7 of ceiling culture of SAT **(C)** and VAT **(D)** samples, day 12 of SAT **(E)** and VAT **(F)** samples, day 20 of SAT **(G)** and VAT **(H)** samples. Photos were taken using the Evolution™ QEi Camera (Media Cybernetics, Rockville, Maryland) attached to the Olympus motorized inverted microscope IX81 (Olympus Corporation, Tokyo, Japan) with a 5x magnification. Bar represent 50 µm.

We first characterized APC in standard culture condition using TEM (Figures 1A,B). These cells displayed visible large nuclei and, in their cytoplasm, multiple small lipid droplets were visible. In comparison, DFAT cells cultivated in ceiling culture on ACLAR after 7 days are presented in Figures 1C–I. Here, the number of small lipid droplets in their cytoplasm was remarkably higher as shown in Figures 1C,E,G These lipid droplets were also visible using ORO (Figure 2). In addition, DFAT cells also showed an invaginated nucleus (Figures 1C,E,G) and slightly enlarged mitochondria, with misaligned cristae (visible in Figure 1H). Some DFAT cells also exhibited an “empty pocket” in their cytoplasm as shown in Figure 1I. Finally, Figure 1F illustrates how DFAT cells were able to secrete collagen fibers as a number of filaments can be seen in the extra-cellular matrix (ECM) (see letter C, for collagen, on Figure 1F).

**FIGURE 3:**
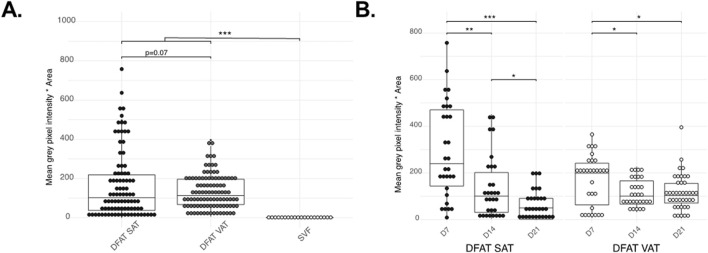
Quantification of the Oil Red O staining. Differences between the cell populations tested (SAT DFAT, VAT DFAT and APC), all time-points confounded (Planned-contrast ANOVA p < 0.001) **(A)**, changes in ORO accumulation in DFAT SAT and VAT during the time points tested **(B)**. Graphs show individual points as well as the boxplot. *P < 0.05 **p < 0.01 ***P < 0.001.

The temporal evolution of the intracellular small lipid droplets in DFAT cells was further investigated using ORO staining. Indeed, we noticed that unilocular adipocytes (Figures 2C,D) become progressively less present, leaving room for multilocular fibroblast-like cells (Figures 2E–H **)**. Interestingly, even though APC possess small intracellular lipid droplets, intracellular lipid accumulation is considerably lower than that observed in DFAT cells (p < 0.001) (Figure 3A). Over time, the intracellular lipid content in DFAT cells diminished and this more rapidly in SAT than VAT DFAT cells (Figure 3B).

**FIGURE 4:**
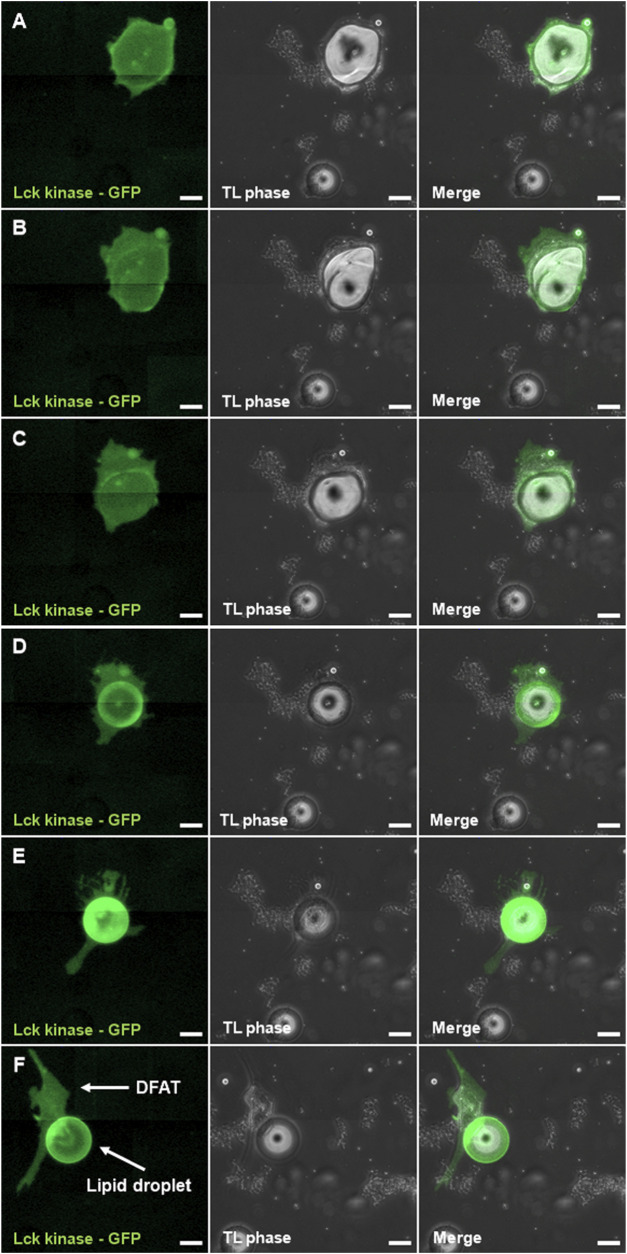
Presence of a membrane around the liposecreted lipid droplet. Sections **(A–D)** follow liposecretion of a mature adipocyte **(A)** all the way through its phase as an almond-shaped cell **(B)**, liposecretion **(C)** and the origin of a fibroblast-like cell (i.e., DFAT cell) and a lipid droplet with a membrane **(D)**. Detail of a fibroblast-like cell and its lipid droplet **(E–F)**. Panels to the left show the channel with Lck-kinase GFP, middle panels show phase contrast, and panels to the right show merged images between the GFP channel and phase contrast. Bar represent 50 µm.

In addition to the characterization of DFAT cells presented above, we studied the phenomena of liposecretion. More specifically, we investigated the presence of a membrane surrounding the ejected lipid droplet. As can be seen in Figure 4 the mature adipocyte (Figure 4A) undergoing liposecretion (Figures 4B,C) ejects a large lipid droplet surrounded by a membrane (Figure 4D). This phenomenon is clearly seen in Figure 4E where both the DFAT cell membrane and the lipid droplet express green fluorescence. To support the changes occurring in lipid droplet morphology, we conducted a secondary analysis of published data by our group (Côté et al., 2017a). Particularly, we investigated cell death inducing DFFA like effector c (CIDEC), a lipid droplet-associated protein involved in lipid droplet growth and implicated in adipocyte energy store adaptations (Gong et al., 2011) and found that CIDEC expression is suppressed before and after liposecretion.

Our last objective was to relate liposecretion to the progression of the cell cycle. Four inhibitors were chosen, based on the phase of the cell cycle that they target: AraC (phase S), Vincristine (phase M), Irinotecan (checkpoint between phases S and G2) and RO-3306 (checkpoint between phases G2 and M). Based on our experiments on APC (details in Supplementary Material), we selected the following concentrations: 0.5 μM AraC, 2 nM Vincristine, 0.5 μM Irinotecan and 5 μM RO-3306 and a maximum time of exposure of 72 h. These conditions were tested using cell count (Supplementary Figure S1), WST-1 cell proliferation assay (Supplementary Figure S2) and CFU (Supplementary Figure S3). Our data suggest that these conditions do not reduce severely the number of living cells. However, caution should be used in the analysis of data with vincristine.

**FIGURE 5:**
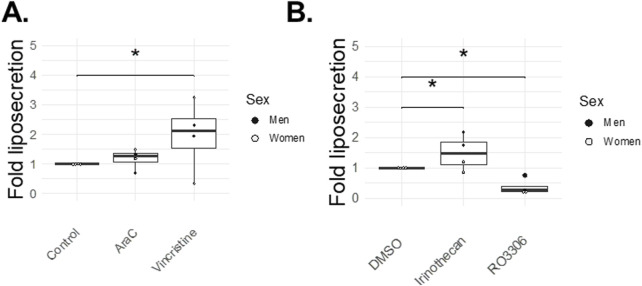
Cell cycle inhibitor effects on liposecretion (72 h). At day three post-isolation, adipocytes in ceiling cultures were incubated with 0.5 μM AraC, 2 nM Vincristine, 0.5 μM Irinothecan, 5 μM RO-3306, 5 μM DMSO (Vehicle) or 20% MAM (Control) for 72 h. Figures illustrate the fold over control (95% confidence interval for Irinothecan: 0.27–0.97, p-value = 0.04; and for RO-3306: −1.1 – −0.16, p = 0.01) **(A)** or fold over vehicle (95% confidence interval for AraC: −0.74–1.11, p-value = NS; and for Vincristine: 0.04–1.89, p-value = 0.04) **(B)** of the liposecretion phenomenon (number of events normalized over total number of adipocytes). Graphs report individual points [n = 4 for each drugs, 2 women (white dots), 2 men (black dots)].

Figure 5 summarizes the effects of the four cell cycle inhibitors on liposecretion. Vincristine increased the occurrence of liposecretion by 1.97-fold over control (p = 0.04) and AraC only had mild effects on liposecretion with a 1.18-fold, non-significant increase over control (Figure 5A), whereas Irinotecan increased liposecretion by 1.50-fold over vehicle (p = 0.04) (Figure 5B). Interestingly, liposecretion seemed to be hindered by the presence of CDK1 inhibitor RO-3306 (Figure 5B), as its occurrence dropped to 0.37-fold over vehicle (p = 0.01).

**FIGURE 6:**
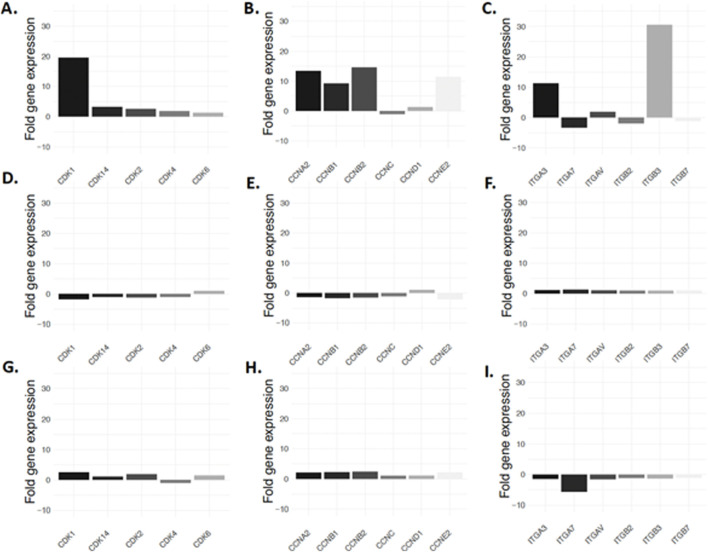
Expression of cell cycle regulators during adipocyte dedifferentiation. Expression of cyclin dependent kinases before liposecretion **(A)**, in the early DFAT stage **(B)** and late DFAT stage **(C)** is illustrated. Cyclines are seen in panels **(D)**, **(E, F)** and integrins in panel **(G–I)**. Data showed display a secondary analysis of the data published by Côté et al. (2017a).

Considering the link between adipocyte dedifferentiation and CDK1, we decided to conduct a secondary analysis of our previously published data (Côté et al., 2017a). Our results illustrate that among several cyclin-dependent kinases, CDK1 is expressed 20 times more than average (19.6 vs. 0.91) before liposecretion (Figure 6A). Interestingly, both cyclin A2 and cyclin B1 which are upregulated during the S phase of the cell cycle and the G2 phase respectively, were also more expressed (Figure 6D). Because recent literature suggests that CDK1 is involved in integrin adhesion and cell cycle related cellular adhesion (Kamranvar et al., 2022), we investigated the expression of integrins. Figure 6G shows an increased expression specifically in integrin 3 (alpha and beta chain) after 4 days of ceiling culture. Such upregulation occurred specifically in the early phase of adipocyte dedifferentiation (Figures 6B,C,E,F,H,I).

## Discussion

Several studies explored the use of DFAT cells to support transplant and organ regeneration (Mimatsu et al., 2023; Murata et al., 2022; Hidaka et al., 2022), as reviewed in (Côté et al., 2019b). Here, we describe both the phenomenon leading to DFAT cells, i.e., liposecretion, and some of the features of the DFAT cell population. Based on our results, DFAT cells are characterized by a significant accumulation of small intracellular lipid droplets that diminish in number over time without completely fading away. They display nuclei invaginations and elongated mitochondria. The DFAT cell population is generated through liposecretion, corresponding to the ejection of a large lipid droplet representing more than 50% of the adipocyte diameter, coated by a membrane.

In line with previous studies (Maurizi et al., 2017; Côté et al., 2019a; Côté et al., 2019b), we confirmed the secretion of a large lipid droplet that does not have any nucleus. Secondly, we showed that the large lipid droplet, after dedifferentiation, is covered by a membrane, as shown in the original report by Maurizi and colleagues and supported by others (Kim et al., 2022), who showed by transmission electron microscopy the presence of a three-layered membrane coating the lipid droplet (Maurizi et al., 2017).

Previous studies conducted by Maurizi and collaborators (Maurizi et al., 2017) suggested that, in ceiling culture, mature adipocytes shed their lipids not trough asymmetrical cell division or lipolysis but rather through liposecretion (Maurizi et al., 2017). Here, we show that the remaining fibroblast-like cell (DFAT cell) does not become lipid-free but rather stores multiple smaller lipid droplets that are partially lost over time. In addition, the analysis of time-lapse images suggested that the phenomenon of liposecretion (Maurizi et al., 2017; Côté et al., 2019a; Côté et al., 2019b) peaks after 5 days post-isolation, which is congruent with a change in their transcriptome (Côté et al., 2017a). Additionally, we show that two of the main human adipose tissue depots, being abdominal subcutaneous (SAT) and abdominal visceral adipose tissue (VAT) can dedifferentiate. However, time course experiments using ORO staining to measure intracellular lipid enrichment suggest that unlike abdominal SAT, abdominal VAT retains significantly more small intracellular lipid droplets compared to control APC. Although additional studies are needed to elucidate the reasons underlying the phenomenon, previous published data show that adipocyte identity genes involved in lipolysis (hormone-sensitive lipase, LIPE), lipogenesis (lipoprotein lipase, LPL; diglyceride acyltransferase, DGAT2) and adipocyte function (ADIPOQ, PLIN1, FABP4) are consistently downregulated during adipocyte dedifferentiation (Côté et al., 2017a), while some (alpha SMA, MMP3) but not all APC markers (PREF1) are increased (not shown). Taken together, these results suggest a change in cellular identity, with a loss of lipolytic capacity in DFAT. Differences in the degree to which these changes occur in SAT versus VAT may explain the intracellular lipid enrichment observed.

We further characterized the DFAT population and compared it to APC using TEM. We confirmed the presence of newly deposited collagen surrounding the DFAT cell population, supporting what was already reported by our group (Côté et al., 2017a; Côté et al., 2017b). Our data draw attention to the presence of long, enlarged mitochondria in both cell populations. This suggest that mitochondria might undergo fusion, a process allowing them to join and increase their performance in oxidative phosphorylation (Adebayo et al., 2021). In the literature, this process is known to be associated with increased cellular stress and ROS production (Adebayo et al., 2021) as well as autophagy (Deleyto-Seldas and Efeyan, 2021). Because our samples were isolated from adipose tissue of donors with severe obesity, it is possible that lipid peroxidation in hypertrophied adipocytes and disbalanced redox state (Cinti, 2023) may have reduced the respiratory capacity and increased the likelihood of mitochondrial fusion. On the other hand, one study identified autophagy as a possible mechanism through which mature adipocytes may undergo dedifferentiation *in vitro* (Pan et al., 2021). The authors showed that by treating adipocytes with rapamycin, a mTOR blocker, and therefore blocking cellular autophagy accelerated the phenomena of dedifferentiation (Pan et al., 2021). Although our study did not investigate the molecular pathways explaining the generation of long, enlarged mitochondria, the literature offers possible mechanisms needing further exploration to explain this phenomenon. We additionally report the presence of nucleus invaginations in DFAT cells. Although nuclei invaginations have already been reported for other cell lines, such as 3T3 cells (Clubb and Locke, 1998), their presence has yet to be reported in DFAT cells. The presence of invagination is also known to emerge in the presence of stiff and abundant ECM (Srivastava et al., 2021), or in senescent cells (Pathak et al., 2021). Senescent cells are additionally characterized by an irreversible arrest of the cell cycle and the inability to proliferate (Campisi, 2013). The possibility that DFAT cells may indeed be a senescent population cannot be excluded, as we repeatedly fail to observe DFAT cells divide during our time-lapse imaging experiments. In addition, recent data gathered in mice by our laboratory showed that the so-called “DFAT population” is indeed significantly contaminated by APC, which can take over the culture relatively more rapidly compared to DFAT cells (Gauthier et al., 2025).

Finally, we studied whether liposecretion is dependent on cell cycle progression. Because previous studies by our group suggested the activation genes related to the cell cycle (Côté et al., 2017a; Côté et al., 2019a), and a large number of publications, including pioneer studies in the field of DFAT cells (Sugihara et al., 1986; Sugihara et al., 1987), reported increased DNA synthesis during adipocyte dedifferentiation, we tested whether incubation with various cell cycle inhibitors was able to prevent or increase adipocyte dedifferentiation. Our data showed a three-fold decrease in the occurrence of liposecretion following the treatment with RO-3306. Such a significant drop in the frequency of liposecretion events was not observed with the other cell cycle inhibitors used, suggesting a potential role of CDK1 in liposecretion signalling. The importance of CDK1 was also demonstrated by the significant increase in CDK1, cyclin A2 and B1 as well as integrin 3 expression before liposecretion occurs. During the last two decades, a number of authors showed how CDK1, by interacting with cyclin A2, participates in the building of canonical cellular adhesion and actin patch which stabilizes the cell during interphase (Jones et al., 2018; Jones and Jones, 2024; Kamranvar et al., 2022; Robertson et al., 2015). The number and densities of these patches change during the cell cycle, increasing during the S phase, reaching its maxima during the G2 phase and rapidly dissolving in the M phase. Related to this topic, CDK1 is also known to have a role in controlling the actin cytoskeleton (McCusker et al., 2012; Karpova et al., 1998). Interestingly, Kim et al. have demonstrated that the actin remodeling involving intracellular actin-mediated force generation, was an important contributor to adipocyte dedifferentiation (Kim et al., 2022). The group also reported the presence of an “actin hole” generated around the liposecreted lipid droplet (Kim et al., 2022), which is consistent with the empty pockets found in some DFAT cells in TEM images. Additional studies are required to validate our hypothesis of the implication of CDK1, intracellular cytoskeleton, cell-ECM connections and integrin signaling in the liposecretion process.

Current literature suggests that adipocyte dedifferentiation may be connected to cellular adhesion and the ECM like Liu and colleagues who proposed a role in ECM remodeling (Liu et al., 2022). This is supported by an independent group (Karanfil et al., 2023) and early work from our group (Lessard et al., 2015b; Côté et al., 2017a; Côté et al., 2017b), suggesting that the phenomenon of dedifferentiation might exist *in vivo* (Wang and Scherer, 2019; Wang et al., 2018), but is exacerbated *in vitro* due to the environmental changes. Although numerous authors suggest the role of cellular adhesion as a driver for liposecretion, to date, no data exist on the role of the cell cycle. Here, we suggest that liposecretion is a cell cycle-dependent phenomenon driven by CDK1, probably playing a role in integrin signaling and the creation of several and more stable cell-ECM connections. Cell cycle-dependent cellular adhesion is likely to play a major role in adipocyte dedifferentiation. Indeed, our study shows that stopping the cell in early S phase using AraC (pyrimidine analog) does not affect liposecretion. On the other hand, stopping the cell when cellular adhesion is increased such as in late S/early G2 phase using Irinotecan (topoisomerase I inhibitor) or is yet to be compromised, like before the M phase with vincristine (inhibition of microtubule polymerization), has the effect of increasing the frequency of liposecretion.

Our study has limitations. Because of their complexity in a primary cell culture context, the number of samples tested in time-lapse experiments is limited and includes donors with large age and BMI ranges, which significantly increases variability. Consequently, we suggest that our results on liposecretion should be interpreted with caution. Exploratory expression experiments would also require validation at the protein level. Finally, our group has recently investigated the possible contamination of the DFAT cell population by APC during collagenase isolation (Gauthier et al., 2025). The presence of contaminating APC cannot be excluded, especially in the analyses of TEM images or ORO DFAT cells.

In conclusion, our study suggests that liposecretion in human adipocytes occurs at a relatively low frequency (1% of adipocytes in culture) and might be cell-cycle related and under control of CDK1. The phenomenon of liposecretion is characterized by the rapid secretion of a large lipid droplet covered with a membrane. Dedifferentiated adipocytes have long, enlarged mitochondria and may retain small lipid droplets for long periods of time.

## Data Availability

The original contributions presented in the study are included in the article/Supplementary Material, further inquiries can be directed to the corresponding author.
