# Supplementary material for: Exploring the role of cell cycle regulation in human mature adipocyte dedifferentiation
Source: Front Cell Dev Biol. 2025 May 8;13:1547836. doi: 10.3389/fcell.2025.1547836 (PMC12095275; doi:10.3389/fcell.2025.1547836)
Supplement: Supplementary file 1 [file Table1.docx]

**Table S1**: Summary of the literature search filed on PubMed in 2019

| **Family** | **Targeted phase** | **Inhibitor** | **Advantages** | **Inconvenient** | **References (PMID)** |
| --- | --- | --- | --- | --- | --- |
| DNA polymerase inhibitors | S | Cytarabine (Ara-C) | Already used in Côté et al. 2019 |  | 30561036 |
| Microtubule inhibitors | M | Vincristine | Already used in Côté et al. 2019 |  | 30561036 |
| Topoisomerase inhibitors | S/G2 | Doxorubicin | 42% survival after 15 days culture at 100ng/mL | Strong apoptosis within 5 days (-50%); some studies show a short effect (24h) | 10413306  26169986  26631614  17893511  32384733 |
| Topoisomerase inhibitors | S/G2 | Irinothecan | Highly used, apoptosis only 9% after 48h at 5uM, stops cell cycle and cellular proliferation within 48h | Proliferation might restart after 72h if concentrations were low. | 31438997  10778989  11986787  18182997  12361906 |
| CDK1 inhibitors/ blockers of Wee1 inhibitors | G2/M | Terameprocol (M4N) | Low toxicity, tested on many cell lines, cell cycle stopped after 72h at 40uM. Arrested cells showed 95% viability after 8 days drug exposure | Apoptosis at 50uM | 11485827  15329416  11454698 |
| CDK1 inhibitors/ blockers of Wee1 inhibitors | G2/M | RO-3306 | Largely used in cell cycle synchronization. | Experiments mostly done with max 72h incubation, longer exposures are associated with toxicity or cellular compensation (increase CDK1 expression) | 31235824 3054512 30045664 24374180 26423135 |
| CDK1 inhibitors/ blockers of Wee1 inhibitors | G2/M | MTH-3 | Cell cycle arrest in 24h | Tested in only one cell line, only one paper published | 29138806 |
| CDK1 inhibitors/ blockers of Wee1 inhibitors | G2/M | Norwogonin | Viability is compromised at 40 uM, cell cycle is stopped after 72h | No effect in non-tumorous cells, only one paper published | 30826569 |

We chose Cytarabine and Vincristine as our team already tested both inhibitors in our cell culture system. We then added Irinothecan (higher viability compared to Doxorubicin) and RO-3306 (high viability, more widely used, tested on multiple cell lines).
