# Supplementary material for: Exploring the role of cell cycle regulation in human mature adipocyte dedifferentiation
Source: Front Cell Dev Biol. 2025 May 8;13:1547836. doi: 10.3389/fcell.2025.1547836 (PMC12095275; doi:10.3389/fcell.2025.1547836)
Supplement: Supplementary file 2 [file DataSheet1.docx]

**SUPPLEMENTARY DATA**

**Supplementary methods:**  The following techniques were used to assess the optimal concentration to be used for each cell cycle inhibitor.

Cell proliferation:

Cells were seeded in a 12-well plate at 20% confluence and incubated with the drugs for 120 hours. To estimate proliferation, cells were counted every 24 hours. Statistical analyses were conducted in R version 4.3.1. Following the assessment of normality and test for homoscedasticity, Kruskal-Wallis non-parametric test was applied followed by post-hoc test (Bonferroni), were used to assess significance.

Toxicity:

Cells were seeded in a 96-well plate at 20% confluence and incubated with the drugs for 120 hours. To estimate toxicity, the medium was tested every 24 hours using ToxiLight™ (Lonza, Maryland, USA) assay. Positive control was with 10% NP-40 diluted in DMEM-F12. Briefly, 20 μL of supernatant was sampled and 25 μL of adenylate kinase detection reagent was added to the detection plate. Luminescence was then measured using Gen 5 Plate Reader (Agilent Technologies, formely BioTek Instruments, Winooski, Vermont, USA). Statistical analyses were conducted in R version 4.3.1. Following the assessment of normality and test for homoscedasticity, Kruskal-Wallis non-parametric test was applied followed by post-hoc test (Bonferroni), were used to assess significance.

Colony forming unit (CFU) assay:

Cells were seeded at 100 cells/cm^2^ and with either 0.5 μM AraC, 2 nM Vincristine, 0.5 μM Irinotecan, 5 μM RO-3306, 5 μM DMSO (Vehicle) or DMEM-F12 (Control) for 72 hours. Cells were then washed with PBS, fixed with 10% formalin for 15 minutes and washed again. Cells were then stained with 1% crystal violet (15 minutes, room temperature). Staining excesses were washed with water. Plates were imaged using Zeiss Axio Observer Z1 Microscope and Axiocam 506 (Zeiss, Oberkochen, Germany). A colony was defined by a minimal diameter of 2 mm with at least 40 cells (Côté et al., 2019a). Statistical analyses were conducted in R version 4.3.1. Following the assessment of normality and test for homoscedasticity, Kruskal-Wallis non-parametric test was applied followed by post-hoc test (Bonferroni), were used to assess significance.

WST-1 Assay cell proliferation:

10 000 cells were seeded with either 0.5 μM AraC, 2 nM Vincristine, 0.5 μM Irinotecan, 5 μM RO-3306 or DMEM-F12 (control). After 72 hours, 10 μL of WST-1 reagent was added and cells were incubated at 37°C, 5% CO_2_ for 2 hours as per manufacturer instructions (Abcam, Waltham Massachusetts, USA). Statistical analyses were conducted in R version 4.3.1. Following the assessment of normality and test for homoscedasticity, Kruskal-Wallis non-parametric test was applied followed by post-hoc test (Bonferroni), were used to assess significance.


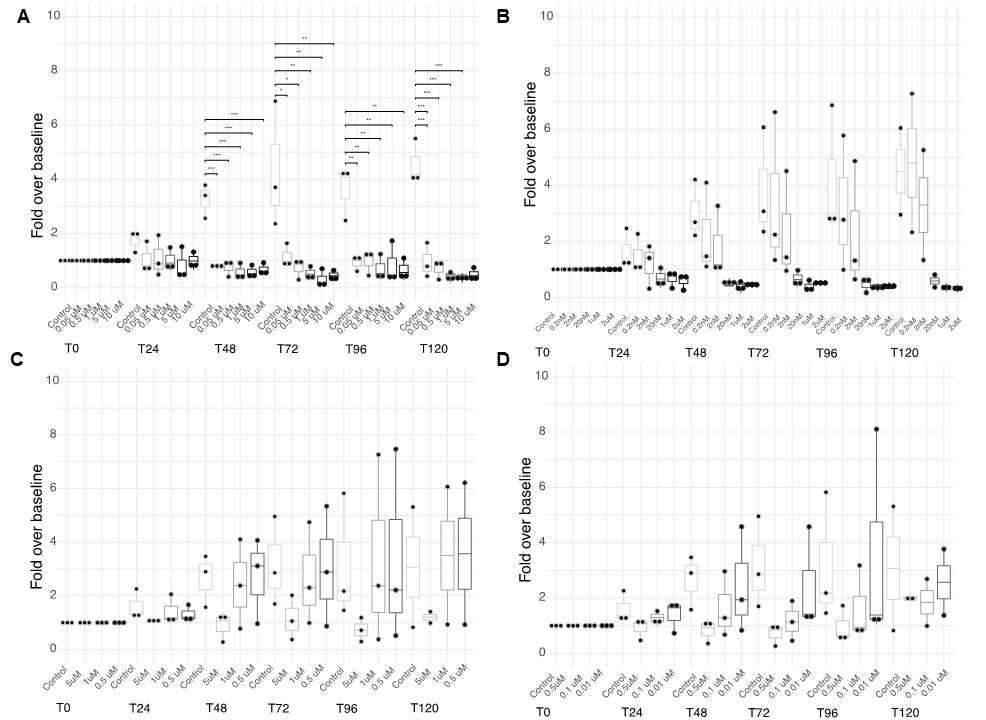


**Supplemental Figure 1: Cell proliferation with or without cell cycle inhibitors (120 hours).** Cells were incubated with 10 μM, 5 μM, 1 μM, 0.5 μM, 0.05 μM AraC (A); 2 μM, 1 μM, 20 nM, 2 nM, 0.2 nM Vincristine (B), 0.5 μM, 0.1 μM, 0.01 μM Irinothecan (C) or 5 μM, 1 μM, 0.5 μM RO-3306 (D) 5 μM DMSO (Vehicle) or DMEM-F12 (Control) up to 120 hours. Cell proliferation was measured every 24 hours. Data are expressed as mean±SD. *p<0.05 **p<0.01 ***p<0.001


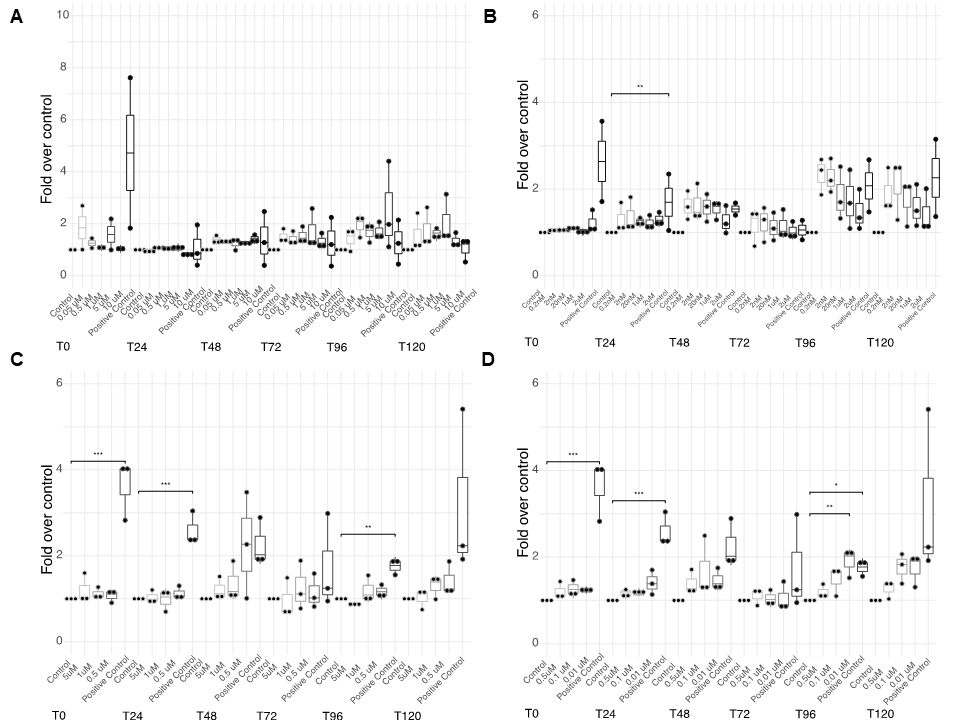


**Supplemental Figure 2: Cell cycle inhibitor toxicity (120 hours).** Cells were incubated with 10 μM, 5 μM, 1 μM, 0.5 μM, 0.05 μM AraC (A); 2 μM, 1 μM, 20 nM, 2 nM, 0.2 nM Vincristine (B), 0.5 μM, 0.1 μM, 0.01 μM Irinothecan (C) or 5 μM, 1 μM, 0.5 μM RO-3306 (D) or 5 μM DMSO (Vehicle) or DMEM-F12 (Control) up to 120 hours. Adenylate kinase release in the medium was measured by fluorimetry every 24 hours. Data are expressed as mean±SD. *p<0.05 **p<0.01 ***p<0.001


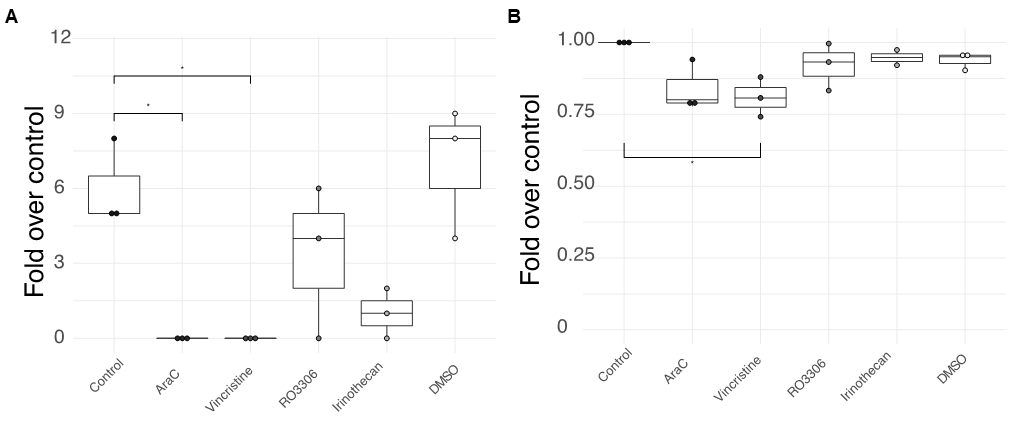


**Supplemental Figure 3: Cell viability with or without cell cycle inhibitors (72 hours).** Cells were incubated with 0.5 μM AraC, 2 nM Vincristine, 0.5 μM Irinothecan, 5 μM RO-3306, 5 μM DMSO (Vehicle) or DMEM-F12 (Control) for 72 hours. Figures illustrate the results for the WST-1 assay (**A**) and the Colony Forming Unit assay (**B**). Data are expressed as mean ±SD. *p<0.05 **p<0.01 ***p<0.
